# Mobile health (mHealth) technologies for fall prevention among older adults in low-middle income countries: bibliometrics, network analysis and integrative review

**DOI:** 10.3389/fdgth.2025.1559570

**Published:** 2025-03-28

**Authors:** Michael Joseph Dino, Ladda Thiamwong, Rui Xie, Ma. Kristina Malacas, Rommel Hernandez, Patrick Tracy Balbin, Joseph Carlo Vital, Jenica Ana Rivero, Vivien Wu Xi

**Affiliations:** ^1^ College of Nursing, University of Central Florida, Orlando, FL, United States; ^2^ Research Development and Innovation Center, Our Lady of Fatima University, Valenzuela, Philippines; ^3^ School of Nursing, Southern Institute of Technology, Invercargill, New Zealand; ^4^ Alice Lee Centre for Nursing Studies, National University of Singapore, Singapore

**Keywords:** mHealth, fall prevention, fall risk, older adults, low-middle income countries, bibliometrics, network analysis, integrative review

## Abstract

**Introduction:**

mHealth technologies offer promising solutions to reduce the incidence of falls among older adults. Unfortunately, publications on their application to Low-Middle Income Countries (LMIC) settings have not been collectively examined.

**Methods:**

A triadic research design involving bibliometrics, network analysis, and model-based integrative review was conducted to process articles (*n* = 22) from 629 publications extracted from major databases using keywords related to mHealth, falls prevention, and LMIC. The web-based application Covidence and stand-alone VosViewer software were used to process data following previously published review standards.

**Results:**

Published articles in the field feature multidisciplinary authorships from multiple scholars in the domains of health and technology. Network analysis revealed the most prominent stakeholders and keyword clusters related to mHealth technology features and applications in healthcare. The papers predominantly focused on the development of mHealth technology, usability, and affordances and less on the physiologic and sociologic attributes of technology use. mHealth technologies in low and middle-income countries are mostly smartphone-based, static, and include features for home care settings with fall detection accuracy of 86%–99.62%. Mixed reality-based mobile applications have not yet been explored.

**Conclusion:**

Overall, key findings and information from the articles highlight a gradually advancing research domain. Outcomes reinforce the need to expand the focus of mHealth investigations to include emerging technologies, update current technology models, create a more human-centered technology design, test mHealth technologies in the clinical setting, and encourage continued cooperation between and among researchers from various fields and environments.

## 1 Introduction

The world is rapidly aging at an unprecedented rate, with declining fertility and mortality rates compared to previous years (1, 2). By 2050, the older adult population will comprise 22% of the global population, and 80% can be found in low- and middle-income countries (LMICs) (2, 3). Despite heightened efforts to increase healthcare investments, LMICs continue to struggle with vulnerable and resource-constrained health and social care systems in supporting the health of the aging population to maintain optimum health while reducing age-related challenges and physical decline (4, 5).

In the older adult population, falls remain a significant public health concern globally (6). A fall is described as an event causing a person to come to rest accidentally on the ground, floor, or similar lower level (7, 8). Its high prevalence in the demographic stems from several predisposing factors, such as advancing age, fear of falling, reduced mobility, and gait problems (9, 10). These conditions adversely affect health, leading to disability and even mortality (11, 12). About 28%–35% of older adults experience falls each year globally (13, 14), making it a challenging global health emergency (6, 15, 16).

Interestingly, it was discovered that older adults residing in LMICs are more susceptible to falls, given the lack of accessible facilities and treatment for chronic conditions that increase the risk for falls (17, 18). In the Philippines, for example, about 18% of older adults experience falls due to lack of proper guidance, unconducive living situations, impaired functional status, presence of physical pain, and limited mobility and strength (6).

The incidence of falls is considered preventable (19) through early assessment and effective interventions such as determination of fall risk factors (20, 21), assessment of the risk for fall-related injuries (22), and alleviation of the fear of falling (23). Prevention methods are considered the pillars of fall management, encompassing targeted physical activities such as balance training (24) and physical therapy exercises (25). Health promotion through fall education (26) and environmental modifications such as smart devices installed at homes (27, 28) or attached to bodies (29, 30) to monitor the health of individuals at risk are some emerging solutions to address fall incidence. In addition, there are also multifactorial interventions (31) such as medication management (32), psychological interventions (33), and assistive devices (34, 35). Fall prevention management strategies must be research-based (36) and integrated with technology to address fall-related challenges effectively (37).

One promising and emerging solution to address fall incidences among community-dwelling older adults is using mobile devices (mHealth) to deliver effective healthcare services to patients remotely (38, 39). mHealth encompasses various remote healthcare processes, services, and technologies, including telemedicine (40), medication adherence (41), fitness (42), and gero-technologies for fall detection and prevention (43–46). Due to their popularity, especially in LMICs, mobile phone or smartphone applications are typically used in mHealth (47, 48). Still, other devices, such as smartwatches (49) and other wearable devices (50), are increasingly being popularized.

The application of mHealth in fall prevention is evident in various levels and specific health conditions or diseases (51). At the primary level, mHealth for falls is effective in promoting health education (52) and assisting with older adults' physical activity through strength and balance training (43) and traditional exercises such as Tai Chi (53). At the secondary level, studies have also proven the usefulness of mHealth for falls in preventive screenings and medication management (54), cognitive assessments (55), and fall detection and alert using home cameras (56). Finally, at the tertiary level, there is evidence of the effectiveness of mHealth during patient recovery post hip surgery (57), bone fractures (58), and management of chronic pain in arthritis among older adults (59). mHealth technologies provide an efficient platform for effective monitoring, health education, and communication that empowers older adults to take a more proactive role in their overall health management promoting better functional independence.

The extent of literature regarding mHealth and its usefulness in fall detection and prevention is widely acknowledged, as evidenced by a wide range of studies published in healthcare and technology. Unfortunately, there is a scarcity of studies that review, map, and assimilate mHealth literature related to fall prevention among older adults, specifically in LMICs where a greater density of the graying population can be found. Therefore, this study addresses this oversight by examining the published literature focusing on LMIC's application of mHealth for falls via bibliometric analysis, mapping, and integrative review. The triangulation of these approaches offers a promising lens for a comprehensive understanding of the growth, status, current landscape, and trajectory of mHealth technology for policy development, quality assurance, and practice improvement.

## 2 The Hamm framework

**Figure 1:**
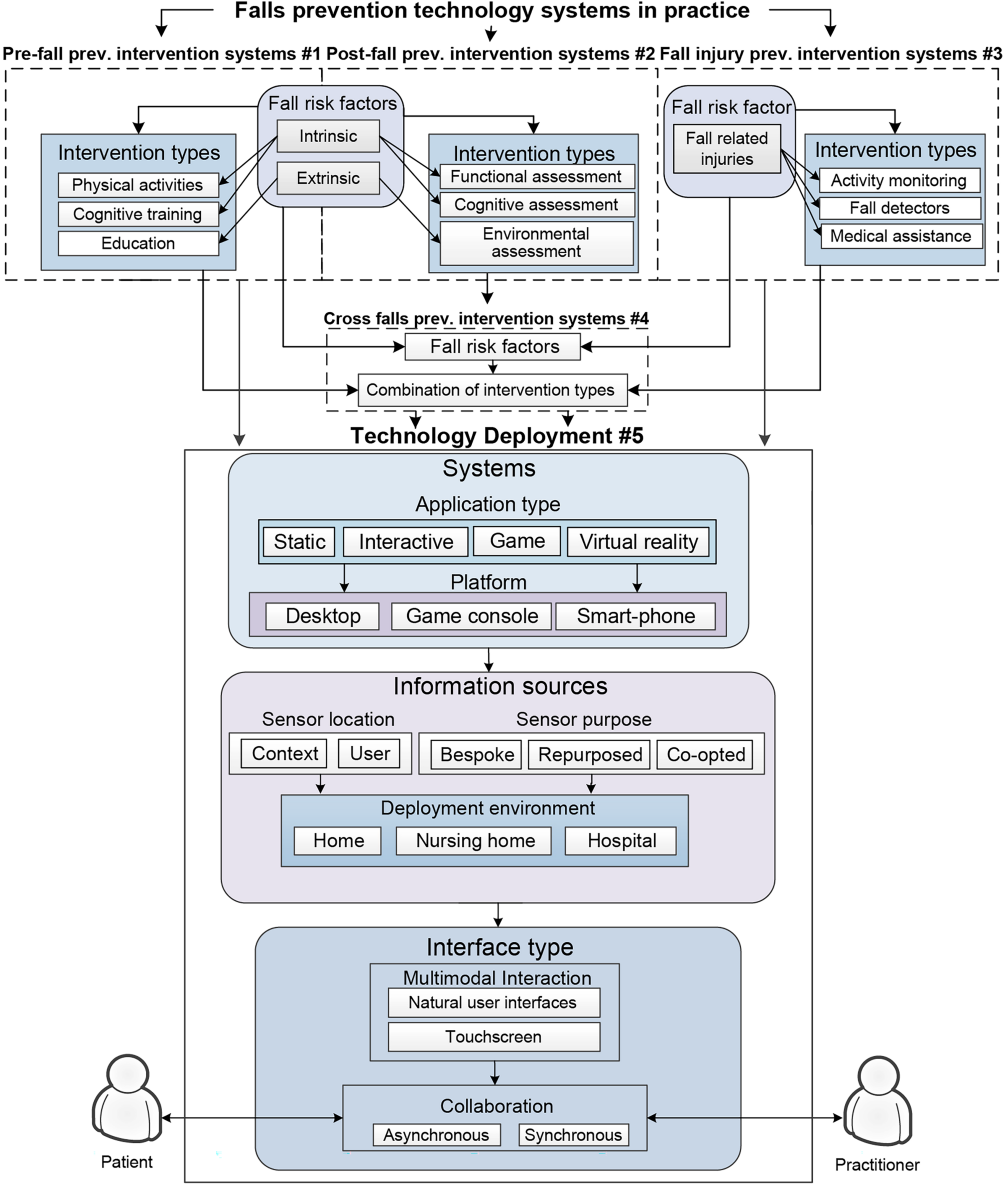
Hamm et al. (60) Conceptual model of fall prevention technology.

The conceptual framework proposed by Hamm et al. (60) provides a systematic approach to evaluating technology systems applied in fall prevention. The framework (Figure 1) is formulated with emphasis on five key interconnected components: pre-fall prevention, post-fall prevention, fall injury prevention, cross-fall prevention, and technology deployment. Pre-fall prevention interventions (Pre-FPIs) center on proactive support for older adults at risk of falling but without a history of falls, such as physical activity exercises, education programs, and cognitive training. Post-fall prevention intervention systems (Post-FPIs) focus on assessing and delivering interventions to older adults who have experienced falls to limit recurrent falls. These interventions include diagnostic assessments (e.g., functional and cognitive assessments) and environmental inspection to assess for external risks that can hinder older adults' function and independence. Systems under the fall injury prevention intervention (FIPIs) target older adults with a high probability of experiencing falls. Interventions in this category include systems to detect falls to prevent further injuries, activity monitoring, and medical assistance once a fall is detected. Technologies designed in combination with either of these systems are categorized as cross-fall prevention intervention systems (CFPIs). Technology development consolidates the application types, platforms, information sources, deployment environment, interface type, and collaboration.

Recent studies have increasingly used the Hamm framework for understanding fall prevention technologies and their features, thus demonstrating its relevance in addressing the multifaceted nature of fall risks among older adults. The framework emphasizes technology integration and focuses on personalized interventions, which various empirical studies have supported. According to the proponents, technological interventions for fall prevention facilitate multiple purposes ranging from diagnostics to fall injury management, as shown in several literature reviews using the framework (61–63). Empirical studies have also utilized the framework to guide interventions focused on critical areas in fall prevention: exercise interventions, fall risk assessments, education interventions, and home assessments (64, 65). Similarly, related studies complement the framework (66–68) in providing a basis for the importance of fall efficacy, user perspectives, and engagement in fall prevention strategies. Congruent, although broader in scope, is the conceptual framework for Computer-Mediated Reality Technologies (CMRT) mapped by Ibrahim and Money (69), as it parallels the adoption of technologies in healthcare contexts using patient-centered design and systems.

In this study, the Hamm et al. (60) framework provided a structure for mapping existing literature and categorizing mHealth applications for fall prevention among older adults in LMICs. Various mHealth applications for falls were categorized depending on their purpose: prevention (Pre-FPIs), assessment (Post-FPIs), detection and response (FIPIs), and combination (CFPIs). Each of these systems was further analyzed depending on their application type (static, interactive, game-based, virtual reality applications), platform focus (desktop, game console, smart-phone), information sources based on sensor location (context, user), sensor purpose (bespoke, repurposed, co-opted), deployment environment (home, nursing home, hospital), interface type (natural user interfaces, touchscreen), and collaboration (asynchronous, synchronous). This framework also systematically analyzed patterns and specific challenges centered on mHealth applications for fall prevention among older adults in LMICs. Results obtained using the model shall guide future research directions to contextualize specific caveats of mHealth technologies tailored to the chosen demographic within the LMIC ecosystem.

## 3 Methods

Health informationist-assisted literature search of various databases - Scopus, Web of Science, PubMed, and Embase – was conducted using a combination of keywords and Boolean expressions related to “mHealth”, “mobile health technologies”, “falls”, “fall detection”, and “lower-to-middle-income countries”, and “LMICs”. The eligibility criteria include studies that are (1) theoretical or empirical, (2) published in the English language, (3) focused on mHealth technologies intended for older adult falls, and (4) conducted at LMICs from the Organization for Economic Co-operation and Development (OECD) listing. Studies were excluded if they fall into any of the following categories: (1) literature review, (systematic or narrative reviews), (2) PhD or Master's Theses, (3) non-peer-reviewed articles, (4) non-English publications, or (5) if full-text papers were unavailable. The quality of theoretical papers was assessed through the 6-scale Authority, Accuracy, Coverage, Objectivity, Date, and Significance (AACODS) checklist (70). In contrast, empirical (research) papers were evaluated using the Mixed Methods Appraisal Tool (MMAT) percentage-based scale (71).

### 3.1 Article bibliometrics and distribution

The articles were categorized based on authorship (i.e., single, double, and multiple), article type (i.e., theoretical/non-research, empirical/research), publication sources (i.e., technology/informatics, health, and health technology, health technology), geographical location according to the WHO classification, and publication year. The articles were also categorized according to their quality appraisal scores, purposes, and critical findings. Furthermore, a map is created through Adobe Illustrator to visually showcase the studies and their origin using the color spectrum technique, where light colors represent areas with fewer studies, and areas shaded with more solid colors indicate greater publication density (72).

### 3.2 Network analysis

Bibliographic coupling network analysis was utilized to cluster the extant publications and analyze mHealth technologies intended for falls among older adults in LMICs. This method is suitable for researchers to visualize the publication landscape of a field effectively (73, 74).

### 3.3 Integrative review

The articles on mHealth for fall prevention of older adults from LMICs were also examined using an integrative review approach. Using published guidelines and article benchmarks (75–78), the study adhered to a five-step process covering (1) problem identification, (2) literature search, (3) data evaluation, (4) data analysis, and (5) presentation. As mentioned, this study also used the Hamm et al. (60) framework to categorize the full-text papers using the following criteria: (1) Technology Systems in Practice, (2) intervention types, and (3) Technology deployment. The technology deployment section is further divided into three subsections: (1) the systems used, which includes the application type and platform; (2) the sources of information, addressing the location, purpose, and deployment environment of the sensors; and (3) the type of interface used, encompassing the multimodal interface and collaboration aspects of the mHealth technology. These labels (Table 1) provide a better understanding of the vital features and characteristics of the mHealth technologies available in LMICs.

**Table 1:** Attributes, categories, and types/features of fall prevention technologies (Hamm framework).

Attributes	Categories	Types	Icon/Label
System	Application	Static	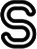
		Interactive	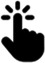
		Game	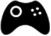
		VR	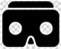
	Platform	Desktop	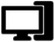
		Game console	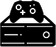
		Smartphone	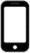
		Smartwatch	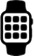
		Sensor	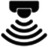
		Others (Cane)	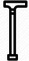
Information sources	Sensor location	Context	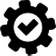
		User	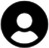
	Purpose	Bespoke	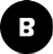
		Repurposed	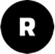
		Co-opted	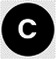
	Deployment	Home	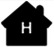
		Nursing home	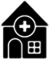
		Hospital	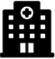
Interface	Multimodal interaction	Natural	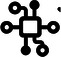
		Touchscreen	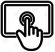
	Collaboration	Asynchronous	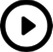
		Synchronous	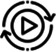

## 4 Results

**Figure 2:**
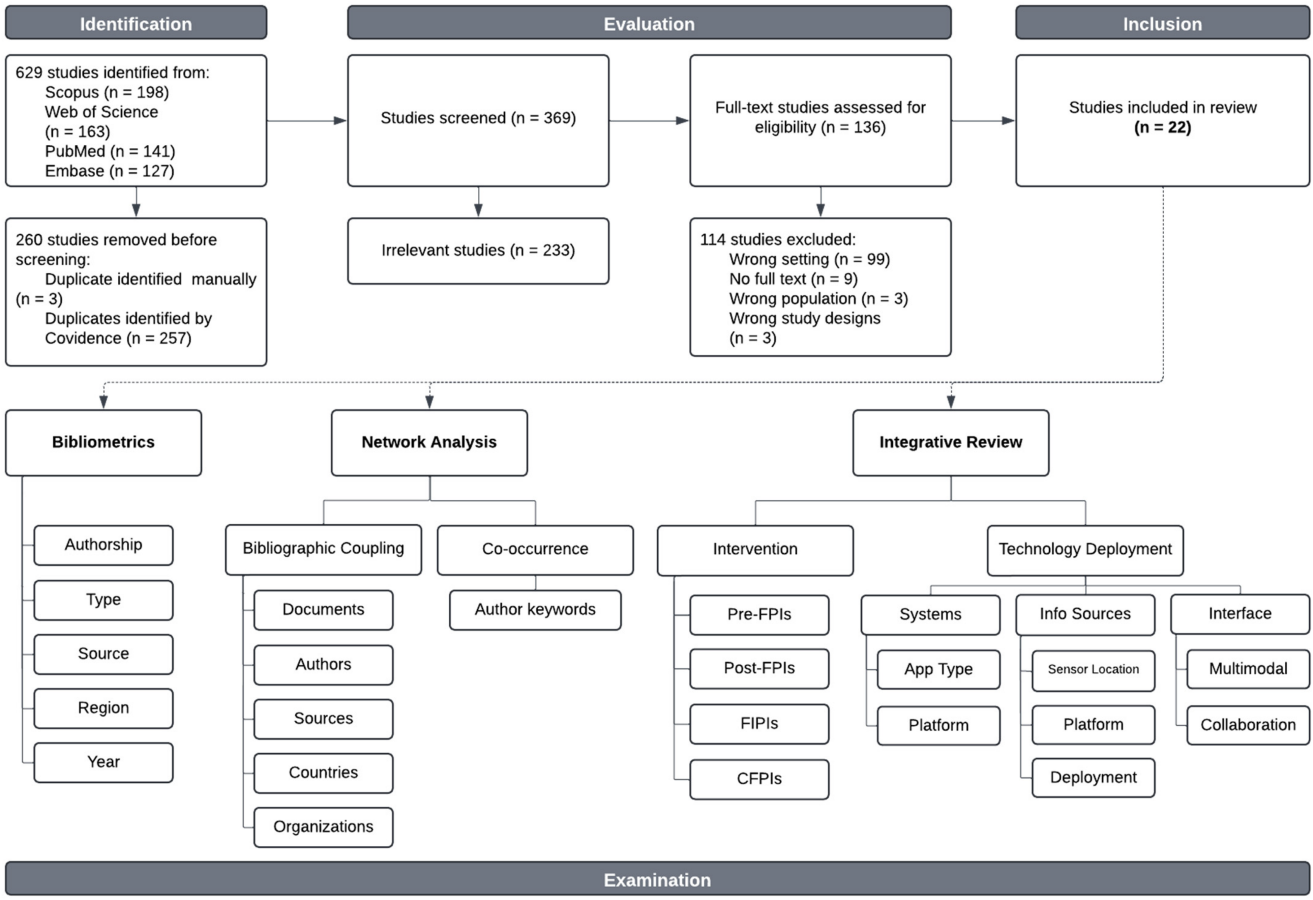
Modified PRISMA flowchart incorporating bibliometrics & network analysis.

A total of 639 publications were generated, uploaded, and screened using the review platform Covidence. After the removal of duplicates (*n* = 260) and irrelevant studies (*n* = 233), a total of 136 articles were advanced for full-text screening. The final articles (*n* = 22) were exported into a CSV file from the Covidence application to extract the relevant data for analysis: citation, bibliographical information, abstract and keywords, funding details, and other article information. The qualified papers were published from 2014 to 2024 and consisted of papers from scholarly conferences (*n* = 14), peer-reviewed articles in academic journals (*n* = 7), and a book chapter (*n* = 1). The VOSViewer application version 1.6.20 (79) was used for bibliographic mapping and visualization of intuitive maps. The software calculates and classifies citation frequencies, number of documents, occurrences, links, and link strength based on parameters such as top participating authors, countries, organizations, journals, and the most prominent keywords and their respective clusters (Figure 2).

### 4.1 Article bibliometrics

The bibliometric characteristics of the 22 articles are shown in Table 2. Most articles have multiple authors (*n* = 17; 77.27%), while almost a quarter of the documents hold partner authorship (*n* = 5; 22.73%). Most articles are tagged as empirical (*n* = 15; 68.18%), while a small portion was theoretical (*n* = 7; 31.82%). There is a preponderance of articles coming from technology informatics journals (*n* = 17; 77.27%), compared to health (*n* = 2; 9.09%) and health informatics (*n* = 3; 13.64%) publications. As to article sources, 13 articles (61.90%) came from Southeast Asia, with minimal documents coming from the Mediterranean (*n* = 5; 23.81%), America (*n* = 2; 9.52%), and Western Pacific (*n* = 1; 4.76%). Articles related to mHealth technologies in LMICs have been limited since 2014, with notable increases in 2022 (*n* = 5; 22.73%) and 2023 (*n* = 7; 31.82%).

**Table 2:** Article bibliometrics.

Attributes	*n*	%
Authorship		
Single	0	0.00
Double	5	22.73
Multiple	17	77.27
Type		
Empirical	15	68.18
Theoretical	7	31.82
Publication source		
Technology/Informatics	17	77.27
Health	2	9.09
Health Informatics	3	13.64
Region		
African	0	0.00
America	2	9.52
South-East Asia	13	61.90
European	0	0.00
Eastern Mediterranean	5	23.81
Western Pacific	1	4.76
Year		
2014	1	4.55
2015	0	0.00
2016	1	4.55
2017	2	9.09
2018	1	4.55
2019	1	4.55
2020	1	4.55
2021	1	4.55
2022	5	22.73
2023	7	31.82
2024	2	9.09

### 4.2 Article information, purpose, and key findings

A robust critical appraisal and evaluation of the articles was employed (see Table 3). The outcome reveals that empirical studies demonstrated robust methodological integrity as evidenced by the average MMAT score of 84%, while theoretical articles yielded an acceptable average AACODS score of 5. Assessment of the purpose and key findings of the articles reveal a substantial focus on developing and evaluating technological solutions designed to monitor and safeguard geriatric health and safety. Fall detection technologies reported a high accuracy rate between 86% and 99.62%. Internet of Things (IoT) devices, wearable sensors, and smartphone applications to formulate full-scale monitoring ecosystems combining several functionalities, including vital sign monitoring (e.g., heart rate, blood pressure, temperature, oxygen levels), real-time geospatial tracking, and emergency alert systems are evident.

**Table 3:** Article information, purpose, and key findings.

Author(s), year	Category, type	Quality appraisal^a^^,^^b^	Purpose	Key finding(s)/information
Aakesh et al., 2023 (80)	Empirical	100%	To develop, implement, and assess a wristband device based on ESP-32 and Arduino Nano microcontrollers to improve aged care, lower healthcare costs, and eventually improve older people's well-being, physical status, and independence	The gadget is feasible in assessing the patients’ heart rate, blood oxygen levels, and body temperature. It can also detect falls, track location, and identify sleepwalking cases. It features an SOS visual alarm button that lights up to alert the caretakers and send notification to provider.
Acharya et al., 2016 (81)	Theoretical	4	To showcase an application for provider-patient communication in case of fall or emergencies.	The application features navigation controls, doctor finder app, mind games, and fall detection capabilities.
Ahmed & Kannan, 2022 (150)	Theoretical	5	To propose a secure, privacy-preserving IoT integration with healthcare units to realize a reliable, available, and secure remote-patient monitoring (RPM) system.	The proposed system provides secure RFID-based authentication, end-to-end communications, and privacy protection. It includes a MOTO 360 watch (biosensor | body sensor) with Android wearable OS, a server with REST framework, and a smartphone application to monitor and detect falls, blood pressure, and heart rate.
Ahmed & Kannan, 2023 (82)	Theoretical	5	To describe a system consisting of mobile services, server-side processing, and data collection from sensors or simulation.	Remote Victim Observation (RVO), equipped with IoT and predictive power, can circumvent problems in healthcare facilities and detect potentially life-threatening situations. The system consists of an Android wearable watch (Biosensor | Body sensor), a REST framework server, and a smartphone app to monitor and detect falls, blood pressure, and heart rate.
Barzallo et al., 2019 (83)	Empirical	100%	To propose a non-invasive wireless system that detects freezing and restarts walking using superficial electrical stimulation during an episode among patients with Parkinson's disease.	The results show feasible diagnostic tests for validating the system, such as precision, sensitivity, and specificity.
Bibiana Magdelene et al., 2023 (84)	Theoretical	5	To showcase an IoT application in the healthcare system that continuously monitors health via a wireless monitoring device.	The developed health monitoring system with a fall detection mechanism can be used at hospitals or homes to track health vitals, including older adults’ temperatures and heart rates.
Biswas et al., 2015 (85)	Theoretical	5	To showcase a system for fall detection using accelerometer data, a novel algorithm, and accurate calculations to detect sudden falls in patients.	The system demonstrates a simple and accurate fall detection algorithm for extended application in remote healthcare monitoring.
Doan et al., 2024 (86)	Empirical	100%	To evaluate a comprehensive fall-detection system, develop an algorithm for fall detection for a walking aid and a fall-detection machine learning model.	The developed fall detection system has generated a 99.62% accuracy in detecting falls.
Ghosh & Ghosh, 2023 (87)	Empirical	100%	To develop and evaluate an end-to-end smart home healthcare system for older adults that monitors activity tracks patient location, detects falls, and provides health recommendations through wearable sensors.	The fall detection accuracy of the system is high in the range of 0.903–0.94.
Guner & Albayrak, 2017 (88)	Empirical	100%	To develop and evaluate a system for identifying and reporting accidents of falls in older adults using a TI ez430-Chronos wearable watch.	The magnitude of the 3-axis accelerometer was detected above the threshold, thus successfully detecting a fall.
Jovanov et al., 2023 (89)	Empirical	60%	To develop and assess a mobility suite for longitudinal older adult monitoring and fall risk assessment through a smartphone attached anteriorly to a patient.	The Mobility Suite for smartphones is equipped with an automated 30-second chair stand test (30SCST). This test assesses the risk of future falls and yields significant results. Men are found to have an 86% lower risk of falling. The tool is efficient in assessing fall risk in a cohort with a low risk of falls.
Kadir et al., 2022 (90)	Theoretical	5	To describe the design and development of a system for monitoring the health of older adults, including fall risk.	A cloud-based IoT monitoring system smartphone application was made using the Flutter framework. The application monitors and displays fall data, temperature, heart rate, and oxygen saturation and can alert the user when a fall has occurred.
Liyakathunisa et al., 2022 (91)	Empirical	60%	To develop and test a system for remotely monitoring daily activities, including falls, which utilizes Bidirectional Gated Recurrent Unit (BiGRU) and Gated Recurrent Unit (GRU) deep learning techniques.	Both deep learning techniques provided promising results, but BIGRU provided more accurate monitoring, with accuracies of 98.14% and 99.26% for AAL and mHealth data, respectively.
Megalingam et al., 2014 (92)	Empirical	60%	To describe the design, development, and test of a system for continuous monitoring of home-bound older adults, including tilt and fall detection.	Sensors attached to the bodies of older adults monitor health status and detect falls through an accelerometer. The sensors can notify a caregiver of any untoward incidents.
Nazeer et al., 2023 (93)	Empirical	100%	To propose a human fall prediction system that aims to predict falls from two positions: walk to fall and sit to fall.	The proposed human fall prediction system offers a reliable and cost-effective solution for predicting falls from two positions: walk to fall and sit to fall.
Pandhi & Tiwari, 2022 (94)	Theoretical	5	To showcase a plan for an application design for older adult patients with dementia.	The application contains almost all functionalities that can aid patients, and their families monitor health and safety. Notable features are Fall detection, Medicine Reminder, Location Sharing, a TODO list, and a Way to Home.
Rasheedy et al., 2021 (95)	Empirical	60%	To evaluate the system usability of a self-administered geriatric assessment smartphone application.	Using the developed mHealth application in geriatric care would lead to greater access to consultations at a lower cost.
Sarwar et al., 2024 (96)	Empirical	60%	To introduce and evaluate a robust fall detection and prediction system using the MHEALTH dataset, combining ConvLSTM and Exponential Smoothing Forecasting.	Results indicate the potential for proactive fall prediction using wearable sensors, which could contribute to improved safety and timely assistance for individuals with fall risks.
Syafiqah Mohd Sharif et al., 2023 (97)	Empirical	100%	To develop and evaluate a fall detection system.	The system yielded a 93.33% accuracy for fall detection, while ADL was 86.67%.
Taheri-Kharameh et al., 2022 (151)	Empirical	60%	To describe and assess the development of a mobile application to support self-management of fall risks and client education based on an individual fall risk assessment.	The fall prevention mobile app helps older adults identify their risk for falls and provides risk management through strength and balance exercises.
Veyilazhagan & Bhanumathi, 2018 (98)	Empirical	100%	To develop and evaluate a system that monitors patients with chronic diseases, elevated blood pressure, and older adults at their homes via an Android application.	The system can monitor vital signs efficiently and reduce hospital visits among older adults.
Zia et al., 2020 (99)	Empirical	100%	To develop and pilot a system that collects information about a patient's health and provides health education based on the patient's unique profile.	The multiclass SVM, together with SBMLR systems, achieved the highest accuracy, with a rate of 99.40%, compared to the three other classifiers used.

^a^ Mixed methods appraisal tool (MMAT) score for empirical articles.

^b^ Authority, accuracy, coverage, objectivity, date, significance (AACODS) score for theoretical articles.

Interestingly, the remote monitoring capability of mHealth technologies is a mainstream function that allows caregivers and healthcare providers to assess patients' conditions even at a distance. Key findings also showed a focus on cost-effectiveness and healthcare accessibility, as several systems were targeted at reducing hospital visits and healthcare costs while optimizing quality of care. Outcomes revealed a clear trajectory towards formulating non-invasive, user-friendly technological solutions to enhance older adults' independence while maintaining safety.

### 4.3 Network analysis

Using VOSViewer (version 1.6.20), network analyses were done focusing on (1) research location or country of article origin, (2) organizations and journal sources, and (3) Article keywords to reveal dominant clusters and themes.

#### 4.3.1 Research locations, countries, and network maps

**Figure 3:**
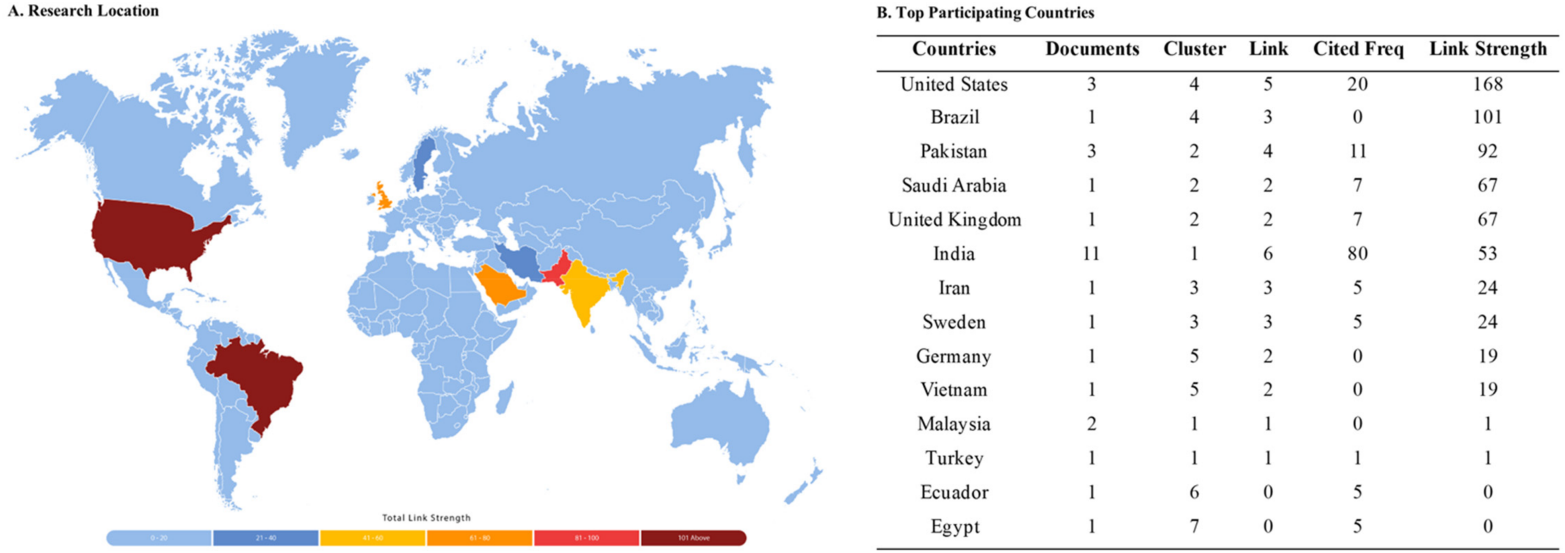
Research locations, and countries of origin.

As shown (Figures 3A,B), five (5) clusters are generated from the articles based on the locations and participating countries. A majority of documents originated from India (*n* = 11; link strength = 53), Pakistan (*n* = 3; link strength = 92), and United States (*n* = 3; link strength = 168). Prominent authors (Table 4) come from only three (3) organizations and have generated a single article with link strengths of either 267 or 403.

**Table 4:** Top participating authors.

Author	Documents	Cluster	Link	Cited Freq	Link strength	Affiliation
Amiri, A.	1	6	11	0	403	University of Alabama, College of Nursing
Bos, A.J.	1	6	11	0	403	Pontifical Catholic University of Rio Grande do Sul
Frith, K.	1	6	11	0	403	University of Alabama, College of Nursing
Jovanov, E.	1	6	11	0	403	University of Alabama, Department of Electrical and Computer Engineering
Oliveira, G.	1	6	11	0	403	Pontifical Catholic University of Rio Grande do Sul
Ahmad, I.	1	8	11	4	267	University of Engineering and Technology, Department of Computer Science & Information Technology
Khalil, W.	1	8	11	4	267	University of Engineering and Technology, Department of Computer Science & Information Technology
Khan, S.	1	8	11	4	267	University of Engineering and Technology, Department of Computer Science & Information Technology
Khan. M	1	8	11	4	267	University of Engineering and Technology, Department of Computer Science & Information Technology
Zia, U	1	8	11	4	267	University of Engineering and Technology, Department of Computer Science & Information Technology

Regarding document-producing organizations, two institutions, the University of Alabama in Alabama, USA, and the Pontifical Catholic University of Rio Grande do Sul in Porto Alegre, Brazil, dominated the list (Table 5) with a link strength of 203. Institutions from Iran and Sweden generated a link strength of 91. All organizations have produced one document each.

**Table 5:** Top document-producing organizations.

Organizations	Location	Documents	Cluster	Link	Cited Freq	Link strength
University of Alabama, College of Nursing	Huntsville, AL, USA	1	6	7	0	203
University of Alabama, Department of Electrical and Computer Engineering	Huntsville, AL, USA	1	6	7	0	203
Pontifical Catholic University of Rio Grande do Sul	Porto Alegre, Brazil	1	6	7	0	203
University of Engineering and Technology, Department of Computer Science and Information Technology	Peshawar, Pakistan	1	7	6	4	134
COMSATS University Islamabad, Department of Computer Science and Information Technology	Islamabad, Pakistan	1	7	6	4	134
Department of Mechanical Engineering, University of Engineering and Technology	Peshawar, Pakistan	1	7	6	4	134
Department of Ergonomics, School of Health	Hamadan, Iran	1	1	7	5	91
Lund University, Department of Health Sciences	Lund, Sweden	1	1	7	5	91
Department of Public Health, School of Health	Hamadan, Iran	1	1	7	5	91
Research Center for Health Sciences	Hamadan, Iran	1	1	7	5	91

The articles originated from various journal publications. The Journal of King Saud University—Computer and Information Sciences and Multimedia Tools and Applications has generated a link strength of 34 each. Notably, most articles were published in information technology and computer science-related journals (Table 6). Only one journal focuses on healthcare: Disability and Rehabilitation: Assistive Technology.

**Table 6:** Top participating journals.

Journals	Documents	Cluster	Link	Cited Freq	Link strength	Journal IF
Journal of King Saud University - Computer and Information Sciences	1	5	1	26	34	5.2
Multimedia Tools and Applications	1	4	1	1	34	3.0
2018 International Conference on Wireless Communications (IEEE)	1	1	2	5	2	23.2
Midwest Symposium on Circuits and Systems (IEEE)	1	1	2	0	2	23.2
Turkish Journal of Electrical Engineering and Computer Science	1	1	2	4	2	1.2
2023 Asia Pacific Conference on Geoscience (IEEE)	1	13	9	0	1	23.2
4th International Conference on Smart Sensors and Application (IEEE)	1	2	1	0	1	23.2
Communications in Computer and Information Science	1	3	1	0	1	0.51
Disability and Rehabilitation: Assistive Technology	1	6	1	5	1	1.9
ICECOM 2016 – Conference Proceedings (IEEE)	1	4	1	1	1	23.2

### 4.4 Article keywords and clusters

**Figure 4:**
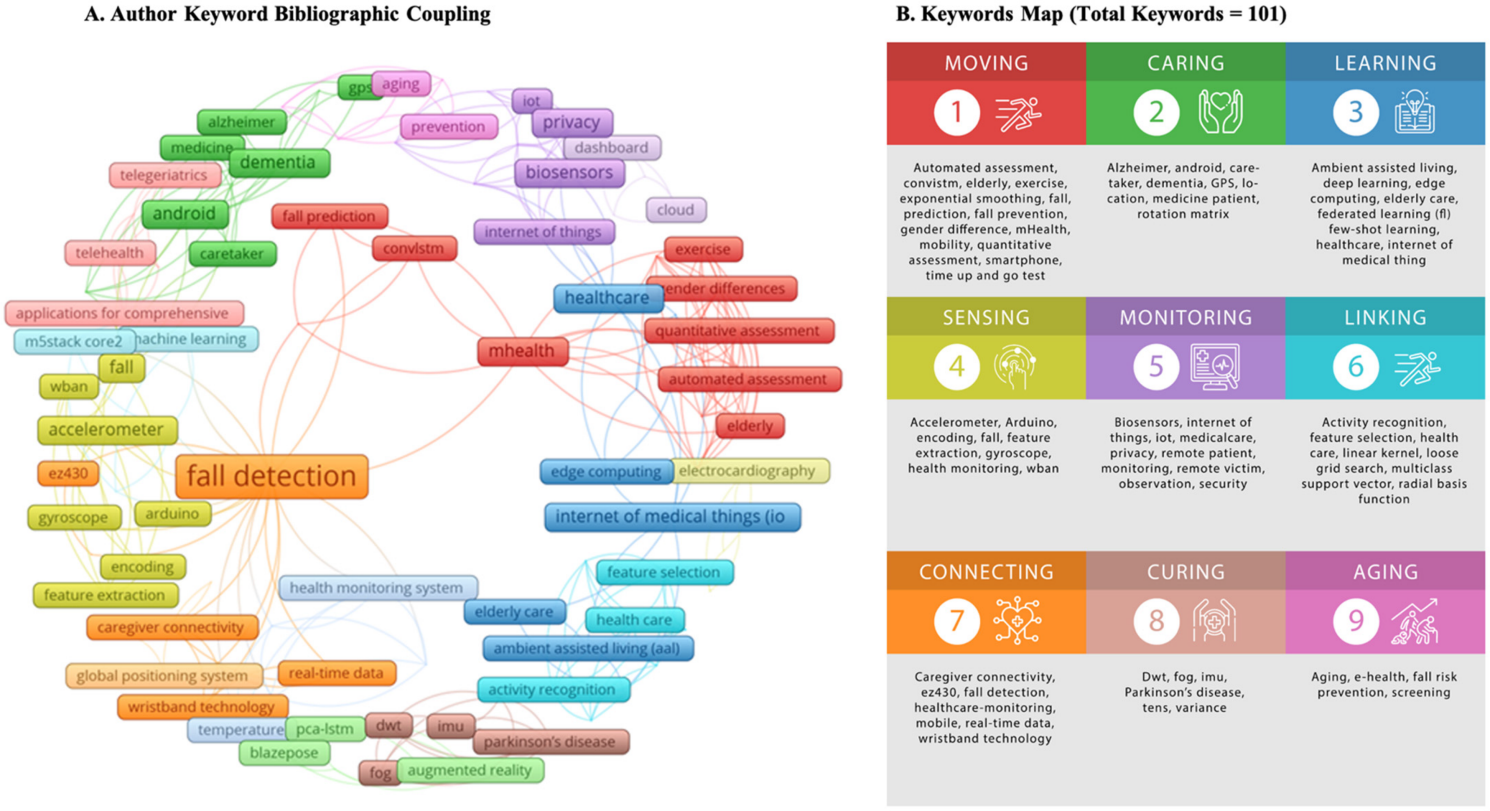
Article keywords and clusters.

“Fall detection” and “mHealth” are the most frequently occurring keywords in the network map (Figure 4A). Nine keyword clusters or groups, tagged as “moving”, “caring”, “learning”, “sensing”, “monitoring”, “linking”, “connecting”, “curing”, and “aging”, were identified from the set of keywords generated by VosViewer (Figure 4B). Most keyword clusters pertain to the technical characteristics of mHealth technologies, as well as the functions and purposes of mHealth technologies for falls and other health conditions.

### 4.5 Typologies and features of mHealth technologies

Table 7 displays the typologies and features of the mHealth technologies featured in the articles based on the attributes presented in the Hamm Framework (65). The prevailing type of technology system used in falls is FIPIs (*n* = 15/22; 68.18%), intended for fall detection (*n* = 16/22; 72.73%). Additionally, nearly all application types are static (*n* = 21/22; 95.45%) and utilize smartphones as their platform (*n* = 16/22; 72.73%).

**Table 7:** Typologies and features of mHealth technologies (hamm framework).

Article: author(s), year	Technology systems in practice	Intervention type (Falls)	Technology deployment						
			Systems		Information sources			Interface type	
			Application	Platform	Sensor	Purpose	Deployment	Interaction	Collaboration
Aakesh et al., 2023 (80)	FIPIs	Detector	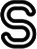	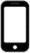	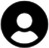	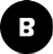	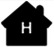	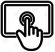	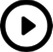
Acharya et al., 2016 (81)	FIPIs	Detector	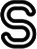	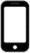	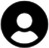	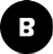	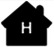	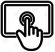	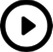
Ahmed & Kannan, 2022 (150)	CFPIs	Detector, Risk assessment	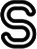	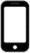	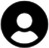	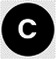	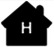	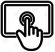	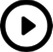
Ahmed & Kannan, 2023 (82)	FIPIs	Detector, Medical assistance	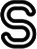	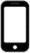	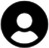	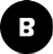	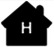	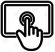	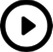
Barzallo et al., 2019 (83)	CFPIs	Functional assessment, Detector, Medical assistance	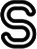	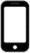	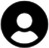	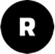	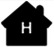	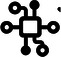	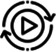
Bibiana Magdelene et al., 2023 (84)	FIPIs	Detector	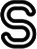	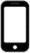	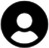	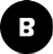	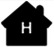	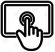	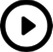
Biswas et al., 2015 (85)	FIPIs	Detector	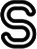	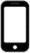	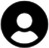	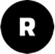	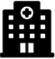	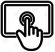	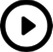
Doan et al., 2024 (86)	FIPIs	Detector	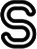	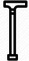	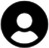	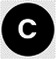	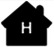	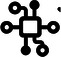	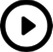
Ghosh & Ghosh, 2023 (87)	FIPIs	Activity monitoring, Detectors, Medical assistance	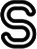	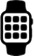	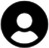	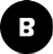	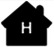	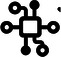	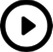
Guner & Albayrak, 2017 (88)	FIPIs	Detector	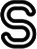	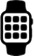	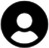	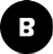	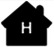	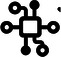	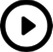
Jovanov et al., 2023 (89)	Pre-FPIs	Physical activity	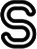	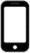	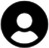	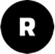	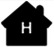	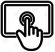	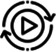
Kadir et al., 2022 (90)	FIPIs	Detector, Activity monitoring	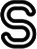	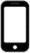	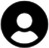	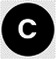	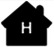	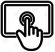	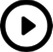
Liyakathunisa et al., 2022 (91)	FIPIs	Detector, Activity monitoring	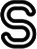	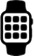 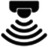	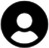	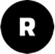 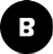	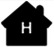	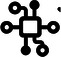	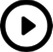
Megalingam et al., 2014 (92)	FIPIs	Detector, Activity monitoring	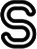	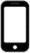 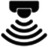	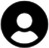	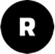 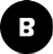	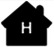	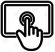	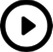
Nazeer et al., 2023 (93)	FIPIs	Detector	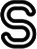	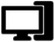	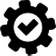	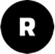	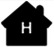	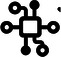	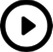
Pandhi & Tiwari, 2022 (94)	CFPIs	Detector	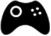	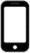	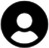	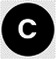	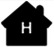	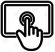	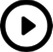
Rasheedy et al., 2021 (95)	Post-FPIs	Functional assessments, Cognitive assessments	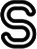	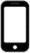	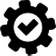	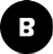	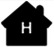	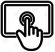	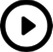
Sarwar et al., 2024 (96)	FIPIs	Detector	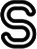	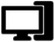	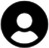	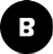	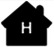	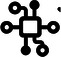	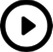
Syafiqah Mohd Sharif et al., 2023 (97)	FIPIs	Detector	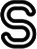	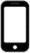	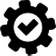 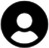	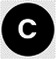	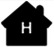	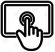	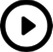
Taheri-Kharameh et al., 2022 (151)	Pre-FPIs	Physical activities, Education	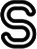	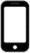	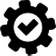	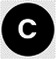	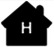	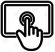	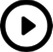
Veyilazhagan & Bhanumathi, 2018 (98)	FIPIs	Detector, Activity monitoring	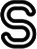	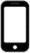	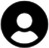	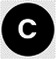	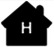	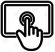	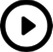
Zia et al., 2020 (99)	Pre-FPIs	Physical activities	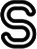	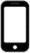	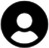	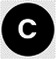	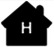	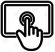	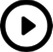

FIPI, fall injury prevention intervention systems; CFPI, cross-fall prevention intervention systems.

Icons: 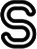 = Static; 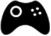 = Game; 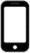 = Smartphone; 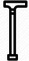 = Cane; 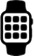 = Smartwatch; 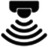 = Sensor; 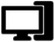 = Desktop; 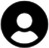 = User; 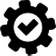 = Context; 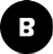 = Bespoke; 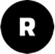 = Repurposed; 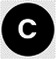 = Co-opted; 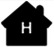 = Home; 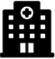 = Hospital; 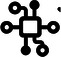 = Natural; 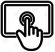 = Touchscreen; 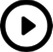 = Asynchronous; 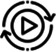 = Synchronous.

Interestingly, some of the articles highlight the use of emerging devices, notably smart watches (*n* = 3/22; 13.64%), desktops (*n* = 2/22; 9.09%), and an intelligent walking cane (*n* = 1/22; 4.55%). Most of the mHealth applications are attached to the user (*n* = 17/22; 77.27%), with the majority falling under bespoke (*n* = 10/22; 45.45%) and co-opted (*n* = 8/22; 36.36%). The interfaces of the mHealth devices are mainly utilizing touchscreen-based interfaces (*n* = 15/22; 68.18%), which are asynchronously used (*n* = 20/22; 90.91%) in a home environment (*n* = 22/22; 100%). Interestingly, there is an absence of virtual reality and interactive mHealth applications for falls. Additionally, no technology has been piloted or tested in nursing homes.

## 5 Discussion

### 5.1 Article bibliometrics

It was found that most articles have multiple authors from various fields, indicating the multidisciplinary nature of mHealth studies for fall prevention among older adults. Projects related to technology applications in health may involve collaborations from private and public stakeholders (100) and healthcare professionals from various practice fields serving diverse clinical populations (101, 102). mHealth solutions to primary care settings are sometimes complex. They may also require expertise from technology personnel to ensure successful implementation (103), particularly among low-income countries where resources must be considered and carefully selected (100). Collaborations in developing and piloting mHealth applications for fall prevention are becoming mainstream and predominant among IT professionals and healthcare scholars. Health researchers collaborating with informaticists and technology engineers can provide practical strategies and quality criteria to optimize mHealth technologies for use among end users such as older adults with fall risks (103–105).

The empirical nature of most mHealth studies bridges technology and healthcare through the postpositivist worldview (106), wherein the potential of mHealth technologies to advance healthcare equates to the quality of mobile interventions formulated through deliberate, empirical research (101). These results emphasize the importance of multidisciplinary collaboration in leveraging technology in fall management to propel healthcare forward, particularly in low-income countries, through data-driven, evidence-based studies.

Notably, both developed and developing nations have their share of scientific studies on mHealth for fall prevention among older adults. Scholars from the Southeast Asia region have led the initiative, alongside developed countries, to benchmark mHealth technologies for fall risk assessment and management. This might be due to fellowships, networks, and interest groups. For instance, the Asia eHealth Information Network, initiated by WHO in 2012, and the Global Digital Health Partnership, launched in 2017 (107), provided avenues for research collaborations among nations. These strategies provide opportunities for professionals to address shared technology challenges and barriers, further advancing mHealth technologies within the region (108) as the last decade has witnessed the emergence of mHealth innovations (109). The technology's expanding usage and advantages have grown dramatically from 2011 to 2020 (110), further fueled by the COVID-19 pandemic (111), and is predicted to continue to grow (112). However, the growth of mHealth publications in LMICs is less active than global trends, as data on mHealth applications between low-income and high-income countries have high discrepancies (110).

### 5.2 Network analysis

India supplies the most significant share of articles in mHealth for older adults in LMICs. The productive scholarship on studies involving older adults runs parallel with the rising older adult population in India. The country's older adult population is projected to grow by 158.67% in 2050, based on a 2019 report (3). Shortly, older adults will constitute the majority of the epidemiologic landscape of India, and the present is the best time to leverage their experiences (113). Furthermore, falls are common in the Indian older adult cohort. A meta-analysis (114) found that 31% of older adults in community settings in India experience falls. Research on older adults and falls in India is growing due to various demographic and health-related factors, even though the country is resource-restrained (115). However, despite landing on the apex, research on older adults and falls in India and other LMICs remains sparse (114).

Nursing, medicine, engineering, information technology, and computer science authors obtained the highest link strengths based on the generated bibliometric maps. Four out of ten authors on the list belong to organizations related to health, and the others belong to computer science and engineering organizations. Health researchers must be equipped with competencies to engage with researchers from other disciplines, such as technology (116), which are drivers of development in healthcare (117). Technology integration into health research is necessary to address the prevailing challenges in health (118), especially with population growth and the rising prevalence of falls among older adults. The inter- and transdisciplinary collaborations between institutions producing technology- and health-related research are encouraged.

The University of Alabama in the USA and the Pontifical Catholic University of Rio Grande do Sul in Porto Alegre, Brazil, occupied the lead spots for document-producing organizations. On the one hand, this result introduces the idea that some technologically advanced countries are interested in conducting mHealth studies for fall prevention for older adults in LMICs. Patients from LMICs commonly experience a plethora of challenges in healthcare due to insufficiencies in infrastructure and finances (119), and higher-income countries that are technologically advanced are willing to provide aid (120). A study (121) emphasized that research strongly correlates with technological advancements in a country. Therefore, technology-based health interventions such as mHealth at LMICs must involve collaboration between the developed and developing world.

The Journal of King Saud University—Computer and Information Sciences and Multimedia Tools and Applications lead publication of mHealth for fall prevention among older adults. Both journals publish studies on technology- and computer science-related research. On the one hand, the Journal of King Saud University—Computer and Information Sciences Journal's commendable publication processing statistics might impact high link strength in the analysis. On the other hand, the Multimedia Tools and Applications journal accepts papers on networked kiosk systems in medicine related to remote technologies such as mHealth. Interestingly, more authors publish mHealth research in technology-related than in health-related journals. There is a need for mHealth researchers to publish health technology journals, especially in gerontology, to attract parallel discussions from both health and technology clusters of experts.

### 5.3 Integrative review

The prevailing type of technology used in falls is the fall and injury prevention intervention systems (FIPIs), primarily purposed for detecting falls. The published literature illustrates a trend toward prioritizing fall injury prevention interventions over pre- and post-fall strategies, such as personal alert systems and remote monitoring strategies (122). The interventions also include reactive systems such as fall injury prevention over more proactive measures such as education and physical exercise activities (123, 124).

mHealth technologies are beneficial in fall detection through alert responses to fall incidents (125, 126). Over the years, mHealth technologies have reduced the number of fall-related injuries in both home and clinical settings (127, 128). Likewise, stakeholders favor immediate fall detection and intervention systems rather than preemptive measures, as they provide immediate alerts and support during fall events (122). The current research landscape is increasingly focused on rapid solutions to prevent fall injuries, highlighting the expanding literature on fall injury prevention systems for older adults. System developers should adopt a long-term approach to interventional systems, encompassing pre- and post-fall strategies.

Nearly all application types are static and adopt smartphones as platforms. This may be due to the high usability and adaptability of smartphones in various scenarios. One noted barrier to adopting mHealth is its complexity (129), and older adults with chronic conditions prefer mHealth systems that are user-friendly and straightforward rather than complex and dynamic. Due to the static nature of most mHealth technologies, older adults are most likely to adopt them because of their seamless functionalities and high usability (130). Additionally, smartphone-based systems are the most used platforms because they effectively monitor and detect falls, given their built-in accelerometers and other sensors. Smartphones are widely preferred in mHealth due to their accessibility, portability, user-friendly interfaces, and ability to combine various healthcare management functions into one device (43, 131). This idea was supported by a study emphasizing the flexibility of smartphones as mobile devices (132). However, the static nature of mHealth technologies for falls investigated in this work might hinder system customizations and personalization necessary for fall sensors to function effectively (133). The static feature of mHealth devices can lead to a one-size-fits-all approach that may only apply to some older adult users. Application developers should develop a system that allows older adults to customize features to provide more tailored and personalized care interventions.

Interestingly, some mHealth technologies mentioned in published studies involve advanced devices such as intelligent walking canes and smartwatches that are not included in the original Hamm Framework of technologies for fall prevention. Integrating emerging mobility devices, such as intelligent walking canes and smartwatches, into fall detection technologies shows potential and promise in enhancing the fall detection capabilities of mHealth systems (134). These devices may offer unique abilities to detect changes in gait and monitor environmental conditions, providing feedback about older adults' stability status and fall risk while in use (135). There is also a growing recognition of advanced measures in fall prevention, most notably the usability of smartwatches (136, 137). Wearable accelerometers incorporated in smartwatches collect accurate data to improve fall prevention for real-time monitoring and alerts, especially where medical assistance is not readily available (138). The machine learning features in smartwatches, integrated with their sensors, promise to provide more inclusive healthcare services (44).

Most of the mHealth application sensors are attached to the user and categorized under bespoke and co-opted typologies. According to Broadley's scoping review (139), most accelerometers for fall prevention are practically connected to users to facilitate immediate feedback. Also, emphasis is placed on the user-based location of sensors because they are more effective than environmental sensors in capturing the dynamic movement of older adults (140). Using lightweight sensors for continuous monitoring, user-centric sensors help identify different types of falls in older adults (141). Bespoke sensors are aligned with the practical design of mHealth applications to address the unique needs of older people. mHealth applications with bespoke sensors are designed to meet optimized fall-detection systems, often applied in nursing homes for custom solutions and seamless workflow for care providers (142). For instance, Okubo et al. (143) found that step training programs tailored for falls reduce fall risk in older adults. Since most mHealth applications are smartphone-based, co-opted purposes of mHealth applications are prevalent. The relevance of co-opted smartphone sensors is helpful in accurately detecting falls through wireless sensor insoles and accelerometers (144). Co-opted smartphone sensors also display their effectiveness with wearable devices and other health applications. Therefore, complementing bespoke devices with co-opted ones provides a comprehensive approach to detecting falls, whereas hybrid solutions offer a more accurate sensor to improve fall strategies (145). Research implies that user-worn sensors, whether purpose-built or smartphone-based, offer promising fall detection strategies and interventions among older adults. Technology developers may adopt customized features of bespoke and co-opted devices due to their comprehensive and accurate nature. However, usability, convenience, and safety must be considered and prioritized for older adults.

The mHealth devices mentioned in the articles mostly use interfaces with touchscreen features, as well as asynchronous and in-home environments. Touchscreen-based interfaces are often used because of their intuitive design and controllability. However, potential challenges related to the physical and sensory limitations of older adults in manipulating touchscreen gadgets must be addressed (146). The development of mHealth interfaces should consider older adults' prevailing comorbidities. Additionally, asynchronous communication features of mHealth devices enhance their feasibility among community-dwelling older adults who need flexibility and convenience in communicating with their healthcare providers (147). Home-based mHealth applications are more conducive to implementation because they can easily be integrated into their daily routines (133). For example, a study emphasized that smartphone-based mHealth interventions are prevalent in reaching community-based older adults who might have difficulties accessing healthcare facilities and programs (148). Also, older adults prefer home-based mHealth technologies because they feel a sense of safety and security, ultimately influencing their preference and use intention for effective mHealth interventions (149).

## 6 Conclusion and recommendation

Health technologies under the mHealth category offer a promising solution to reduce the incidence of falls among older adults. However, literature on their application to LMIC settings has not yet been explored and mapped. The current study examines published articles via bibliometrics, network analysis, and model-based integrative review. Using a web-based application, published papers from major databases were extracted, uploaded, evaluated, and analyzed.

Results show the preponderance of multidisciplinary and multiple authorships from scholars in health and technology domains from developed and developing nations. Collaborations are evident across authors from multiple disciplines, settings, contexts, and working environments, communicating the universality of the topic that interests scholars from territories, even in high-resource settings. Network analysis revealed the most prominent stakeholders and keyword clusters in advancing the science of mHealth technologies for fall prevention among older adults. Previous publications have focused on developing the mHealth technology, its application in fall prevention, usability, and affordances. The integrative review provided a clear picture of the features of commonly used mHealth applications, which are primarily static, smartphone-based, asynchronous, and with features linked with the specific needs of older adult technology users living in home settings. Equally promising mHealth technologies, such as mixed reality applications, have not yet been explored.

The results of this study must be interpreted in recognition of some limitations that might have impacted its outcomes. First, the project utilized peer-reviewed articles that potentially excluded important articles from grey literature that were not indexed in literature databases. Also, the current intent of the study to include only English publications might have prohibited related articles written in foreign languages from being represented in the results. Finally, results of this study only apply to low- and middle-income countries and may not be true to locations with advanced economies.

Overall, the consistent but gradual growth of articles from LMICs in mHealth for fall prevention provided an initial portrait and status of the science in this area of digital health. The mHealth applications for older adults' fall prevention in LMICs are still developing and have not been maximized. Outcomes reinforce the need to update current technology models, typologies, and nomenclatures to include emerging innovations such as mixed reality and their associated mHealth devices. Further studies incorporating variables related to user intention to use, user experience, and physiologic and sociologic attributes of fall prevention using mHealth beyond the technical characteristics of technologies are encouraged. These approaches will encourage healthcare and technology communities to cooperate and co-produce technologies and investigations that are less techno-centric and cognizant of the human-centered design approach.

## Data Availability

The raw data supporting the conclusions of this article will be made available by the authors, without undue reservation.

## References

[1] The Lancet Healthy Longevity. Care for ageing populations globally. Lancet Healthy Longev. (2021) 2(4):e180. 10.1016/S2666-7568(21)00064-7 34697611 PMC8529576

[2] World Population Prospects—Population Division—United Nations. (2024). Available at: https://population.un.org/wpp/ (Accessed October 29, 2024).

[3] United Nations. World Population Ageing 2019 Highlights. New York: United Nations (2019). 10.18356/9df3caed-en

[4] Goodman-Palmer D Ferriolli E Gordon AL Greig C Hirschhorn LR Ogunyemi AO Health and wellbeing of older people in LMICs: a call for research-informed decision making. Lancet Glob Health. (2023) 11(2):e191–2. 10.1016/S2214-109X(22)00546-0 36669801

[5] Kruk ME Gage AD Arsenault C Jordan K Leslie HH Roder-DeWan S High-quality health systems in the sustainable development goals era: time for a revolution. Lancet Glob Health. (2018) 6(11):e1196–252. 10.1016/S2214-109X(18)30386-3 30196093 PMC7734391

[6] Mgabhi PS Chen T-Y Cruz G Vu NC Saito Y. Falls among community-dwelling older adults in the Philippines and Vietnam: results from nationally representative samples. Injury. (2024) 55(3):111336. 10.1016/j.injury.2024.111336 38350305

[7] Hauer K Lamb SE Jorstad EC Todd C Becker C. Systematic review of definitions and methods of measuring falls in randomised controlled fall prevention trials. Age Ageing. (2006) 35(1):5–10. 10.1093/ageing/afi218 16364930

[8] O’Malley N Clifford AM Comber L Coote S. Fall definitions, faller classifications and outcomes used in falls research among people with multiple sclerosis: a systematic review. Disabil Rehabil. (2022) 44(6):855–63. 10.1080/09638288.2020.1786173 32628889

[9] Gale CR Westbury LD Cooper C Dennison EM. Risk factors for incident falls in older men and women: the English longitudinal study of ageing. BMC Geriatr. (2018) 18(1):1–9. 10.1186/s12877-018-0806-3 29769023 PMC5956831

[10] Yeung SSY Reijnierse EM Pham VK Trappenburg MC Lim WK Meskers CGM Sarcopenia and its association with falls and fractures in older adults: a systematic review and meta-analysis. J Cachexia Sarcopenia Muscle. (2019) 10(3):485–500. 10.1002/jcsm.12411 30993881 PMC6596401

[11] Ek S Rizzuto D Xu W Calderón-Larrañaga A Welmer A-K. Predictors for functional decline after an injurious fall: a population-based cohort study. Aging Clin Exp Res. (2021) 33(8):2183–90. 10.1007/s40520-020-01747-1 33161531 PMC8302494

[12] Pinto D Alshahrani M Chapurlat R Chevalley T Dennison E Camargos BM The global approach to rehabilitation following an osteoporotic fragility fracture: a review of the rehabilitation working group of the international osteoporosis foundation (IOF) committee of scientific advisors. Osteoporos Int. (2022) 33(3):527–40. 10.1007/s00198-021-06240-7 35048200

[13] Bergen G Stevens MR Kakara R Burns ER. Understanding modifiable and unmodifiable older adult fall risk factors to create effective prevention strategies. Am J Lifestyle Med. (2021) 15(6):580–9. 10.1177/1559827619880529 34916876 PMC8669903

[14] World Health Organization. (2008). WHO global report on falls prevention in older age. Available at: https://www.who.int/publications/i/item/9789241563536 (Accessed October 29, 2024).

[15] James K Orkaby AR Schwartz AW. Foot examination for older adults. Am J Med. (2021) 134(1):30–5. 10.1016/j.amjmed.2020.07.010 32805226 PMC9614715

[16] Xu Q Ou X Li J. The risk of falls among the aging population: a systematic review and meta-analysis. Front Public Health. (2022) 10:902599. 10.3389/fpubh.2022.902599 36324472 PMC9618649

[17] Barik M Panda SN Tripathy SS Sinha A Ghosal S Acharya AS Is multimorbidity associated with higher risk of falls among older adults in India? BMC Geriatr. (2022) 22(1):486. 10.1186/s12877-022-03158-5 35658840 PMC9167508

[18] Smith J Allen J Haack C Wehrmeyer K Alden K Lund M Impact of app-delivered mindfulness meditation on functional connectivity, mental health, and sleep disturbances among physician assistant students: randomized, wait-list controlled pilot study. JMIR Form Res. (2021) 5(10):1–21. 10.2196/24208 PMC8564666 34665153

[19] Almarwani M. Moving the needle on implementing fall prevention programs in Saudi Arabia: assessing knowledge and perceptions of fall risk among community-dwelling older women. BMC Geriatr. (2024) 24(1):666. 10.1186/s12877-024-05269-7 39118016 PMC11308389

[20] Appeadu M Bordoni B. Falls and Fall Prevention In Older Adults. Treasure Island, FL: National Library of Medicine (2023).

[21] Harper KJ Mast E Carter G Katnich T Oldham V Morrisby C. Prioritising patients for hospital occupational therapy to reduce inpatient falls: a retrospective case-control study to identify predictive patient falls risk factors. Br J Occup Ther. (2023) 86(11):747–54. 10.1177/03080226231181019 PMC12033743 40336791

[22] Hwang JS Kim SH Lee SC Kim S Cho GC Kim MJ Risk factors for ground-level fall injuries during active activity in older patients. Signa Vitae. (2024) 7:85–95. 10.22514/sv.2023.099

[23] Han J Wang H Ding Y Li Q Zhai H He S. Effect of Otago exercise on fear of falling in older adults: a systematic review and meta-analysis. BMC Sports Sci Med Rehabil. (2024) 16(1):132. 10.1186/s13102-024-00917-2 38877578 PMC11177432

[24] Firoz A Azharuddin M Usmani M Parveen S Sehgal CA Noohu MM. Comparison of effects of balance training exercise and gaze stability exercises on balance and postural control in elderly with fall risk: a randomized controlled trial. PhysOccup Ther Geriatr. (2024) 42(3):305–21. 10.1080/02703181.2024.2317730

[25] Pitluk Barash M Shuper Engelhard E Elboim-Gabyzon M. Feasibility and effectiveness of a novel intervention integrating physical therapy exercise and dance movement therapy on fall risk in community-dwelling older women: a randomized pilot study. Healthcare (Switzerland). (2023) 11(8):1–17. 10.3390/healthcare11081104 PMC10137670 37107938

[26] Campos-Mesa MDC Rosendo M Morton K DelCastillo-Andrés Ó. Effects of the implementation of an intervention based on falls education programmes on an older adult population practising pilates–A pilot study. Int J Environ Res Public Health. (2023) 20(2):1–9. 10.3390/ijerph20021246 PMC9859372 36673985

[27] Bui T Liu J Cao J Wei G Zeng Q. Elderly fall detection in Complex environment based on improved YOLOv5s and LSTM. Appl Sci. (2024) 14(19):1–28. 10.3390/app14199028

[28] Kaur N Rani S Kaur S. Real-time video surveillance based human fall detection system using hybrid haar cascade classifier. Multimed Tools Appl. (2024) 83(28):71599–617. 10.1007/s11042-024-18305-w

[29] Sykes ER. An analysis of current fall detection systems and the role of smart devices and machine learning in future systems. Lecture Notes in Networks and Systems, 652 LNNS (2023). p. 502–20. 10.1007/978-3-031-28073-3_36

[30] Wang X Cao J Zhao Q Chen M Luo J Wang H Identifying sensors-based parameters associated with fall risk in community-dwelling older adults: an investigation and interpretation of discriminatory parameters. BMC Geriatr. (2024) 24(1):1–12. 10.1186/s12877-024-04723-w 38302872 PMC10836006

[31] Novaes ADC Ansai JH Alberto SN Caetano MJD Rossi PG de Melo ML Effects of a multifactorial program with case management for falls prevention on functional outcomes in community-dwelling older people: a randomized clinical study. Healthcare. (2024) 12(15):1–11. 10.3390/healthcare12151541 PMC11311896 39120244

[32] Crawford P Plumb R Burns P Flanagan S Parsons C. A quantitative study on the impact of a community falls pharmacist role, on medicines optimisation in older people at risk of falls. BMC Geriatr. (2024) 24(1):1–10. 10.1186/s12877-024-05189-6 39009970 PMC11251379

[33] Drahota A Udell JE Mackenzie H Pugh MT. Psychological and educational interventions for preventing falls in older people living in the community. Cochrane Database Syst Rev. (2024) 2024(10):1–14. 10.1002/14651858.CD013480.pub2 PMC11448480 39360568

[34] Ganga G Arulselvi S. A new intelligent human walking cane type robot. Int J Appl Eng Res. (2014) 9(22):6264–9.

[35] Joseph AM Kian A Begg R. State-of-the-art review on wearable obstacle detection systems developed for assistive technologies and footwear. Sensors. (2023) 23(5):1–31. 10.3390/s23052802 PMC10007677 36905003

[36] Henry A Haddad Y Bergen G. Older adult and healthcare provider beliefs about fall prevention strategies. Am J Lifestyle Med. (2024) 18(1):108–17. 10.1177/15598276221100431 39184271 PMC11339757

[37] Mark JA. The primary care NP’s guide to prevention and management of falls in older adults. Nurse Pract. (2024) 49(2):12–8. 10.1097/01.NPR.0000000000000138 38271144

[38] Manyati TK Mutsau M. A systematic review of the factors that hinder the scale up of mobile health technologies in antenatal care programmes in Sub-Saharan Africa. Afr J Sci Technol Innov Dev. (2021) 13(1):125–31. 10.1080/20421338.2020.1765479

[39] Opoku D Stephani V Quentin W. A realist review of mobile phone-based health interventions for non-communicable disease management in Sub-Saharan Africa. BMC Med. (2017) 15(1):24. 10.1186/s12916-017-0782-z 28162090 PMC5292812

[40] Taha AR Shehadeh M Alshehhi A Altamimi T Housser E Simsekler MCE The integration of mHealth technologies in telemedicine during the COVID-19 era: a cross-sectional study. PLoS One. (2022) 17:1–13. 10.1371/journal.pone.0264436 PMC8870491 35202424

[41] Kgasi M Chimbo B Motsi L. Mhealth self-monitoring model for medicine adherence of patients with diabetes in resource-limited countries: structural equation modeling approach. JMIR Form Res. (2023) 7(1):1–22. 10.2196/49407 PMC10628689 37870902

[42] Wang J-W Zhu Z Shuling Z Fan J Jin Y Gao Z-L Effectiveness of mHealth app–based interventions for increasing physical activity and improving physical fitness in children and adolescents: systematic review and meta-analysis. JMIR Mhealth Uhealth. (2024a) 12:1–27. 10.2196/51478 PMC11094610 38687568

[43] Arkkukangas M Cederbom S Tonkonogi M Umb Carlsson Õ. Older adults’ experiences with mHealth for fall prevention exercise: usability and promotion of behavior change strategies. Physiother Theory Pract. (2021) 37(12):1346–52. 10.1080/09593985.2020.1712753 31910707

[44] De Oliveira FS Da Silva CC Pinheiro TS Yokoi LM Dos Santos PD Tanaka H Assessment of mHealth solutions applied to fall detection for the elderly. In: Blobel B Giacomini M, editors. Studies in Health Technology and Informatics. Amsterdam: IOS Press (2021). p. 239–44. 10.3233/SHTI210606 34734880

[45] Mathew G Bava N Varghese AD Sushan A Benjamin AI. Project vayoraksha: implementation of novel mHealth technology for healthcare delivery during COVID-19 in geriatric population of Kerala. Indian J Med Res. (2024) 159(3 & 4):289–97. 10.25259/IJMR_62_23 39361788 PMC11414785

[46] Oostrik L Holstege M Meesters J Achterberg W Isselt EFVDV. The effects of mHealth in geriatric rehabilitation on health status: a systematic review. Arch Gerontol Geriatr. (2025) 129:1–14. 10.1016/j.archger.2024.105654 39437452

[47] Deepa M Shruti M Mohan V. Chapter 1—reducing the global burden of diabetes using mobile health. In: Klonoff DC Kerr D Mulvaney SA, editors. Diabetes Digital Health. Amsterdam: Elsevier (2020). p. 3–23. 10.1016/B978-0-12-817485-2.00001-8

[48] Osei E Mashamba-Thompson TP. Mobile health applications for disease screening and treatment support in low-and middle-income countries: a narrative review. Heliyon. (2021) 7(3):e06639. 10.1016/j.heliyon.2021.e06639 33869857 PMC8035664

[49] Tumuhimbise W Theuring S Kaggwa F Atukunda EC Rubaihayo J Atwine D Enhancing the implementation and integration of mHealth interventions in resource-limited settings: a scoping review. Implement Sci. (2024) 19(1):72. 10.1186/s13012-024-01400-9 39402567 PMC11476919

[50] Gungormus DB Garcia-Moreno FM Bermudez-Edo M Sánchez-Bermejo L Garrido JL Rodríguez-Fórtiz MJ A semi-automatic mHealth system using wearable devices for identifying pain-related parameters in elderly individuals. Int J Med Inf. (2024) 184:1–7. 10.1016/j.ijmedinf.2024.105371 38335744

[51] van Acker J Maenhout L Compernolle S. Older adults’ user engagement with Mobile health: a systematic review of qualitative and mixed-methods studies. Innov Aging. (2023) 7(2):igad007. 10.1093/geroni/igad007 37007638 PMC10053647

[52] Johnson N Bradley A Klawitter L Johnson J Johnson L Tomkinson GR The impact of a telehealth intervention on activity profiles in older adults during the COVID-19 pandemic: a pilot study. Geriatrics. (2021) 6(3):68. 10.3390/geriatrics6030068 34209416 PMC8293040

[53] Jiraphan J Pensri P Vadhanasin P Lawsirirat C. Benefits of an 8-form tai chi training exercise on balance performance, falling risk, and muscle strengths in elderly with limited strength: a feasibility study. Asian J Appl Sci. (2019) 7:3. 10.24203/ajas.v7i3.5869

[54] Chen P-J Chen K-M Hsu H-F Belcastro F. Types of exercise and training duration on depressive symptoms among older adults in long-term care facilities. Ageing Res Rev. (2022) 77:101613. 10.1016/j.arr.2022.101613 35339704

[55] Li F Harmer P. Prevalence of falls, physical performance, and dual-task cost while walking in older adults at high risk of falling with and without cognitive impairment. Clin Interv Aging. (2020) 15:945–52. 10.2147/CIA.S254764 32606636 PMC7319501

[56] De Miguel K Brunete A Hernando M Gambao E. Home camera-based fall detection system for the elderly. Sensors. (2017) 17(12):2864. 10.3390/s17122864 29232846 PMC5751723

[57] Prieto-Moreno R Estévez-López F Molina-Garcia P Mora-Traverso M Deschamps K Claeys K Activehip+: a feasible mHealth system for the recovery of older adults after hip surgery during the COVID-19 pandemic. Digit Health. (2022) 8:205520762211396. 10.1177/20552076221139694 PMC9677169 36420319

[58] Mora-Traverso M Molina-Garcia P Prieto-Moreno R Borges-Cosic M Cruz Guisado V Del Pino Algarrada R An m-health telerehabilitation and health education program on physical performance in patients with hip fracture and their family caregivers: study protocol for the ActiveHip+ randomized controlled trial. Res Nurs Health. (2022) 45(3):287–99. 10.1002/nur.22218 35148434

[59] Martin CL Bakker CJ Breth MS Gao G Lee K Lee MA The efficacy of mobile health interventions used to manage acute or chronic pain: a systematic review. Res Nurs Health. (2021) 44(1):111–28. 10.1002/nur.22097 33341989

[60] Hamm J Money AG Atwal A Paraskevopoulos I. Fall prevention intervention technologies: a conceptual framework and survey of the state of the art. J Biomed Inform. (2016) 59:319–45. 10.1016/j.jbi.2015.12.013 26773345

[61] Nishchyk A Chen W Pripp AH Bergland A. The effect of mixed reality technologies for falls prevention among older adults: systematic review and meta-analysis. JMIR Aging. (2021) 4(2):e27972. 10.2196/27972 34255643 PMC8280833

[62] Oh-Park M Doan T Dohle C Vermiglio-Kohn V Abdou A. Technology utilization in fall prevention. Am J Phys Med Rehabil. (2021) 100(1):92–9. 10.1097/PHM.0000000000001554 32740053

[63] Schuartz P Ferreira ALA Bernardo LD Raymundo TM Palm RDCM. Occupational therapist’s actions in preventing falls of the elderly person at home: an integrative review of literature (2017–2022). Cad Bras Ter Ocup. (2023) 31:e3526. 10.1590/2526-8910.ctoar270335262

[64] Hamm J Money A Atwal A. Fall prevention self-assessments via mobile 3D visualization technologies: community dwelling older adults’ perceptions of opportunities and challenges. JMIR Hum Factors. (2017) 4(2):e15. 10.2196/humanfactors.7161 28630034 PMC5495970

[65] Hamm J Money AG Atwal A Ghinea G. Mobile three-dimensional visualisation technologies for clinician-led fall prevention assessments. Health Inform J. (2019) 25(3):788–810. 10.1177/1460458217723170 PMC6769285 28816091

[66] Guo X Wang Y Wang L Yang X Yang W Lu Z Effect of a fall prevention strategy for the older patients: a quasi-experimental study. Nurs Open. (2023) 10(2):1116–24. 10.1002/nop2.1379 36178024 PMC9834535

[67] Kwon J Lee Y Young T Squires H Harris J. Qualitative research to inform economic modelling: a case study in older people’s views on implementing the NICE falls prevention guideline. BMC Health Serv Res. (2021) 21(1):1020. 10.1186/s12913-021-07056-1 34583685 PMC8479997

[68] McMahon SK Greene EJ Latham N Peduzzi P Gill TM Bhasin S Engagement of older adults in STRIDE ’s multifactorial fall injury prevention intervention. J Am Geriatr Soc. (2022) 70(11):3116–26. 10.1111/jgs.17983 35924574 PMC9669158

[69] Ibrahim Z Money AG. Computer mediated reality technologies: a conceptual framework and survey of the state of the art in healthcare intervention systems. J Biomed Inform. (2019) 90:103102. 10.1016/j.jbi.2019.103102 30641140

[70] Tyndall J. AACODS Checklist. (2010).

[71] Pluye P Gagnon M-P Griffiths F Johnson-Lafleur J. A scoring system for appraising mixed methods research, and concomitantly appraising qualitative, quantitative and mixed methods primary studies in mixed studies reviews. Int J Nurs Stud. (2009) 46(4):529–46. 10.1016/j.ijnurstu.2009.01.009 19233357

[72] Buda K Cermakova K Hodges HC Fornasiero EF Sukenik S Holehouse AS. Using graphs and charts in scientific figures. Trends Biochem Sci. (2023) 48(11):913–6. 10.1016/j.tibs.2023.08.011 37837963

[73] Hill AM Ross-Adjie G McPhail SM Jacques MBiostat A Bulsara M Cranfield A Incidence and associated risk factors for falls in older adults after elective total knee replacement surgery: a prospective cohort study. Am J Phys Med Rehabil. (2022) 101(5):454–9. 10.1097/PHM.0000000000001848 34292196

[74] Yadav S Chhabra A Mahesh G. Mapping, clustering, and analysis of research in psychiatric genomics. Psychiatr Genet. (2022) 32(6):221–37. 10.1097/YPG.0000000000000325 36302202

[75] Dai F Liu Y Ju M Yang Y. Nursing students’ willingness to work in geriatric care: an integrative review. Nurs Open. (2021) 8(5):2061–77. 10.1002/nop2.726 34388864 PMC8363346

[76] Dino MJS Davidson PM Dion KW Szanton SL Ong IL. Nursing and human-computer interaction in healthcare robots for older people: an integrative review. Int J Nurs Stud Adv. (2022) 4:100072. 10.1016/j.ijnsa.2022.100072 38745638 PMC11080351

[77] Song Y Jung MY Park S Hasnain M Gruss V. Challenges of interprofessional geriatric practice in home care settings: an integrative review. Home Health Care Serv Q. (2023) 42(2):98–123. 10.1080/01621424.2022.2164541 36596311

[78] Whittemore R Knafl K. The integrative review: updated methodology. J Adv Nurs. (2005) 52(5):546–53. 10.1111/j.1365-2648.2005.03621.x 16268861

[79] Van Eck NJ Waltman L. Software survey: VOSviewer, a computer program for bibliometric mapping. Scientometrics. (2010) 84(2):523–38. 10.1007/s11192-009-0146-3 20585380 PMC2883932

[80] Aakesh U Yaswant R Bethanney JJ. Wristband for elderly individuals: esp-32 and arduino nano enabled solution for health monitoring and tracking. 2023 IEEE Asia-Pacific Conference on Geoscience, Electronics and Remote Sensing Technology (AGERS) (2023). p. 26–33. 10.1109/AGERS61027.2023.10491010

[81] Acharya MH Gokani TB Chauhan KN Pandya BP. Android application for dementia patient. 2016 International Conference on Inventive Computation Technologies (ICICT) (2016). p. 1–4. 10.1109/INVENTIVE.2016.7823231

[82] Ahmed MI Kannan G. Safeguards and weightless of electronic chain of command consolidated for virtual patient evaluation. Multimed Tools Appl. (2023) 82(1):453–78. 10.1007/s11042-022-13310-3 35694035 PMC9170562

[83] Barzallo B Punin C Llumiguano C Huerta M. Wireless assistance system during episodes of freezing of gait by means superficial electrical stimulation. In: Lhotska L Sukupova L Lacković I Ibbott GS, editors. World Congress on Medical Physics and Biomedical Engineering 2018. Vol. 68/3. Singapore: Springer (2019). p. 865–70. 10.1007/978-981-10-9023-3_156

[84] Bibiana Magdelene D Jancy S Amutha Mary AV Suji Helen L Selvan MP. Healthcare monitoring system with fall detection mechanism. 2023 Eighth International Conference on Science Technology Engineering and Mathematics (ICONSTEM) (2023). p. 1–5. 10.1109/ICONSTEM56934.2023.10142820

[85] Biswas A Oh PI Faulkner GE Bajaj RR Silver MA Mitchell MS Sedentary time and its association with risk for disease incidence, mortality, and hospitalization in adults: a systematic review and meta-analysis. Ann Intern Med. (2015) 162(2):123–32. 10.7326/M14-1651 25599350

[86] Doan T-N Schroter E Phan T-B. Fall detection using intelligent walking-aids and machine learning methods. In: Thai-Nghe N Do T-N Haddawy P, editors. Intelligent Systems and Data Science. Vol. 1949. Singapore: Springer (2024). p. 95–109. 10.1007/978-981-99-7649-2_8

[87] Ghosh S Ghosh SK. FEEL: FEderated LEarning framework for ELderly healthcare using edge-IoMT. IEEE Transactions on Computational Social Systems. (2023) 10(4):1800–9. 10.1109/TCSS.2022.3233300

[88] Guner H Albayrak Y. System design to detect fall in elderly. 2016 22nd International Conference on Applied Electromagnetics and Communications (ICECOM) (2017). p. 1–3. 10.1109/ICECom.2016.7843899

[89] Jovanov E Oliveira-Zmuda GG Amiri A Bos AJG Frith KH. Assessment of fall risks in older females and males using an automated smartphone mobility suite. In: Principles of Gender-Specific Medicine. Amsterdam: Elsevier (2023). p. 531–49. 10.1016/B978-0-323-88534-8.00033-X

[90] Kadir ADIA Alias MRNM Dzaki DRM Azizan A Din NM. Mobile IoT cloud-based health monitoring dashboard application for the elderly. 2022 4th International Conference on Smart Sensors and Application (ICSSA) (2022). p. 161–6. 10.1109/ICSSA54161.2022.9870913

[91] Liyakathunisa, Alsaeedi A Jabeen S Kolivand H. Ambient assisted living framework for elderly care using internet of medical things, smart sensors, and GRU deep learning techniques. J Ambient Intell Smart Environ. (2022) 14(1):5–23. 10.3233/AIS-210162

[92] Megalingam RK Pocklassery G Jayakrishnan V Mourya G Thulasi AA. Smartphone based continuous monitoring system for home-bound elders and patients. 2014 International Conference on Communication and Signal Processing (2014). p. 1173–7. 10.1109/ICCSP.2014.6950039

[93] Nazeer M Chaitanya Kolliboyina VS Tiruveedula KK Punithavathi IsH Shwetha C Anusha D. Fall prediction of elder person using CCTV footage and Media framework. 2023 International Conference on Emerging Techniques in Computational Intelligence (ICETCI) (2023). p. 138–44. 10.1109/ICETCI58599.2023.10331422

[94] Pandhi S Tiwari R. Dementia care: an android application for assisting dementia patients. 2022 3rd International Conference on Intelligent Engineering and Management (ICIEM) (2022). p. 356–61. 10.1109/ICIEM54221.2022.9853066

[95] Rasheedy D. Mohamed H. E. Saber H. G. Hassanin H. I. (2021). Usability of a self-administered geriatric assessment mHealth: cross-sectional study in a geriatric clinic. Geriatr Gerontol Int, 21(2), 222–8. 10.1111/ggi.14122 33381892

[96] Sarwar MA Chea B Widjaja M Saadeh W. An AI-based approach for accurate fall detection and prediction using wearable sensors. 2024 IEEE 67th International Midwest Symposium on Circuits and Systems (MWSCAS) (2024). p. 118–21. 10.1109/MWSCAS60917.2024.10658849

[97] Syafiqah Mohd Sharif NA Zaki Ayob M Yusoff SB. Development of wearable fall detection alert for elderly. 2023 International Conference on Engineering Technology and Technopreneurship (ICE2T) (2023). p. 311–5. 10.1109/ICE2T58637.2023.10540559

[98] Veyilazhagan R Bhanumathi V. An outdoor intelligent health care patient monitoring system. 2017 International Conference on Innovations in Green Energy and Healthcare Technologies (IGEHT) (2018). p. 1–6. 10.1109/IGEHT.2017.8094061

[99] Zia U Khalil W Khan S Ahmad I Khan MN. Towards human activity recognition for ubiquitous health care using data from awaist-mounted smartphone. Turk J Electr Eng Comput Sci. (2020) 28(2):646–63. 10.3906/elk-1901-31

[100] Capponi G Corrocher N. Patterns of collaboration in mHealth: a network analysis. Technol Forecast Soc Change. (2022) 175:121366. 10.1016/j.techfore.2021.121366

[101] Ben-Zeev D Schueller SM Begale M Duffecy J Kane JM Mohr DC. Strategies for mHealth research: lessons from 3 mobile intervention studies. Adm Policy Ment Health Ment Health Serv Res. (2015) 42(2):157–67. 10.1007/s10488-014-0556-2 PMC4232479 24824311

[102] Gallouj F Rubalcaba L Toivonen M Windrum P. Understanding social innovation in services industries. Ind Innov. (2018) 25(6):551–69. 10.1080/13662716.2017.1419124

[103] Bally ELS Cesuroglu T. Toward integration of mHealth in primary care in The Netherlands: a qualitative analysis of stakeholder perspectives. Front Public Health. (2020) 7:1–17. 10.3389/fpubh.2019.00407 PMC6974538 32010660

[104] Birkhoff SD Moriarty H. Challenges in mobile health app research: strategies for interprofessional researchers. J Interprof Educ Pract. (2020) 19:100325. 10.1016/j.xjep.2020.100325

[105] Montero-Odasso M Van Der Velde N Martin FC Petrovic M Tan MP Ryg J World guidelines for falls prevention and management for older adults: a global initiative. Age Ageing. (2022) 51(9):afac205. 10.1093/ageing/afac205 36178003 PMC9523684

[106] Godinho MA Jonnagaddala J Gudi N Islam R Narasimhan P Liaw S-T. Mhealth for integrated people-centred health services in the western Pacific: a systematic review. Int J Med Inf. (2020) 142:104259. 10.1016/j.ijmedinf.2020.104259 32858339

[107] Singh P Landry M. Harnessing the potential of digital health in the WHO South-East Asia region: sustaining what works, accelerating scale-up and innovating frontier technologies. WHO South East Asia J Public Health. (2019) 8(2):67. 10.4103/2224-3151.264848 31441439

[108] Lwin HNN Punnakitikashem P Thananusak T. E-Health research in Southeast Asia: a bibliometric review. Sustainability. (2023) 15(3):1–19. 10.3390/su15032559

[109] Gutierrez MA Moreno RA Rebelo MS. Chapter 3—information and communication technologies and global health challenges. In: de Fátima Marin H Massad E Gutierrez MA Rodrigues RJ Sigulem D, editors. Global Health Informatics. Amsterdam: Academic Press (2017). p. 50–93. 10.1016/B978-0-12-804591-6.00004-5

[110] Tajudeen FP Bahar N Maw Pin T Saedon NI. Mobile technologies and healthy ageing: a bibliometric analysis on publication trends and knowledge structure of mHealth research for older adults. Int J Hum Comput Interact. (2021) 38(2):118–30. 10.1080/10447318.2021.1926115

[111] McCool J Dobson R Whittaker R Paton C. Mobile health (mHealth) in low- and middle-income countries. Annu Rev Public Health. (2022) 43:525–39. 10.1146/annurev-publhealth-052620-093850 34648368

[112] Galetsi P Katsaliaki K Kumar S. Exploring benefits and ethical challenges in the rise of mHealth (mobile healthcare) technology for the common good: an analysis of mobile applications for health specialists. Technovation. (2023) 121:102598. 10.1016/j.technovation.2022.102598

[113] Ambade M Kim R Subramanian SV. Experience of health care utilization for inpatient and outpatient services among older adults in India. Public Health Pract. (2024) 8:100541. 10.1016/j.puhip.2024.100541 PMC11413678 39309250

[114] Kaur R Kalaivani M Goel AD Goswami AK Nongkynrih B Gupta SK. Burden of falls among elderly persons in India: a systematic review and meta-analysis. Natl Med J India. (2020) 33(4):195–200. 10.4103/0970-258X.316253 34045371

[115] Muneera K Muhammad T Pai M Ahmed W Althaf S. Associations between intrinsic capacity, functional difficulty, and fall outcomes among older adults in India. Sci Rep. (2023) 13(1):9829. 10.1038/s41598-023-37097-x 37330570 PMC10276857

[116] van Baalen S Boon M. Understanding disciplinary perspectives: a framework to develop skills for interdisciplinary research collaborations of medical experts and engineers. BMC Med Educ. (2024) 24(1):1000. 10.1186/s12909-024-05913-1 39272191 PMC11401306

[117] Moen A Chronaki C Petelos E Voulgaraki D Turk E Névéol A. Diversity in health informatics: mentoring and leadership. Public Health and Informatics: Proceedings of MIE 2021 (2021). p. 1031–5. 10.3233/SHTI210341 34042835

[118] Lepore D Dolui K Tomashchuk O Shim H Puri C Li Y Interdisciplinary research unlocking innovative solutions in healthcare. Technovation. (2023) 120:102511. 10.1016/j.technovation.2022.102511

[119] Lopez JCF De Veyra CM Geroy LSA Sales RKP Dizon TS Cutiongco-de La Paz EM. Envisioning the health research system in the Philippines by 2040: a perspective inspired by AmBisyon Natin 2040. Acta Med Philipp. (2019) 53:3. 10.47895/amp.v53i3.148

[120] Liu C Van Wart M Kim S Wang X McCarthy A Ready D. The effects of national cultures on two technologically advanced countries: the case of e-leadership in South Korea and the United States. Aust J Public Admin. (2020) 79(3):298–329. 10.1111/1467-8500.12433

[121] Wang EC. R&D efficiency and economic performance: a cross-country analysis using the stochastic frontier approach. J Policy Model. (2007) 29(2):345–60. 10.1016/j.jpolmod.2006.12.005

[122] Avella-Rodríguez E Gómez L Ramirez-Scarpetta J Rosero E. Colombian stakeholder perceptions and recommendations regarding fall detection systems for older adults. Geriatrics. (2023) 8(3):51. 10.3390/geriatrics8030051 37218831 PMC10204450

[123] Sapci AH Sapci HA. Innovative assisted living tools, remote monitoring technologies, artificial intelligence-driven solutions, and robotic systems for aging societies: systematic review. JMIR Aging. (2019) 2(2):e15429. 10.2196/15429 31782740 PMC6911231

[124] Soh S-E Ayton D Morello R Natora A Yallop S Barker A. Understanding the profile of personal alert Victoria clients who fall. Health Soc Care Community. (2018) 26(5):759–67. 10.1111/hsc.12601 30011101

[125] Changizi M Kaveh MH. Effectiveness of the mHealth technology in improvement of healthy behaviors in an elderly population—a systematic review. mHealth. (2017) 3:51–51. 10.21037/mhealth.2017.08.06 29430455 PMC5803024

[126] Hsieh KL Fanning JT Rogers WA Wood TA Sosnoff JJ. A fall risk mHealth app for older adults: development and usability study. JMIR Aging. (2018) 1(2):e11569. 10.2196/11569 31518234 PMC6716481

[127] Choi NG Choi BY DiNitto DM Marti CN Kunik ME. Fall-related emergency department visits and hospitalizations among community-dwelling older adults: examination of health problems and injury characteristics. BMC Geriatr. (2019) 19(1):303. 10.1186/s12877-019-1329-2 31711437 PMC6849272

[128] Lungu D. Patient safety: a systematic review of the literature with evidence based measures to improve patient safety in healthcare settings. Texila Int J Acad Res. (2023) 10(2):27–35. 10.21522/TIJAR.2014.10.02.Art003

[129] Cajita MI Hodgson NA Lam KW Yoo S Han H-R. Facilitators of and barriers to mHealth adoption in older adults with heart failure. CIN Comput Inform Nurs. (2018) 36(8):376–82. 10.1097/CIN.0000000000000442 29742549 PMC6086749

[130] Best R Souders DJ Charness N Mitzner TL Rogers WA. The role of health Status in older Adults’ perceptions of the usefulness of eHealth technology. In: Zhou J Salvendy G, editors. Human Aspects of IT for the Aged Population. Design for Everyday Life. Vol. 9194. Singapore: Springer International Publishing (2015). p. 3–14. 10.1007/978-3-319-20913-5_1 PMC6592032 31240277

[131] Yoder CLM Kyoshi-Teo H Ochoa-Cosler O. Fall prevention care management: implementation and outcomes of a project to reduce fall risks of older adults in assisted living facilities. J Nurs Care Qual. (2023) 38(4):374–80. 10.1097/NCQ.0000000000000715 37126440

[132] Kim BY Lee J. Smart devices for older adults managing chronic disease: a scoping review. JMIR Mhealth Uhealth. (2017) 5(5):e69. 10.2196/mhealth.7141 28536089 PMC5461419

[133] Kim H Park E Lee S Kim M Park EJ Hong S. Self-Management of chronic diseases among older Korean adults: an mHealth training, protocol, and feasibility study. JMIR Mhealth Uhealth. (2018) 6(6):e147. 10.2196/mhealth.9988 29959109 PMC6045790

[134] Pietrzak E Cotea C Pullman S. Does smart home technology prevent falls in community-dwellingolder adults: a literature review. J Innov Health Inform. (2014) 21(3):105–12. 10.14236/jhi.v21i3.56 25207613

[135] Gill S Seth N Scheme E. A multi-sensor cane can detect changes in gait caused by simulated gait abnormalities and walking terrains. Sensors. (2020) 20(3):631. 10.3390/s20030631 31979224 PMC7038366

[136] Manini TM Mendoza T Battula M Davoudi A Kheirkhahan M Young ME Perception of older adults toward smartwatch technology for assessing pain and related patient-reported outcomes: pilot study. JMIR Mhealth Uhealth. (2019) 7(3):e10044. 10.2196/10044 30912756 PMC6454335

[137] Rimland JM Abraha I Dell’Aquila G Cruz-Jentoft A Soiza R Gudmusson A Effectiveness of non-pharmacological interventions to prevent falls in older people: a systematic overview. The SENATOR project ONTOP series. PLoS One. (2016) 11(8):e0161579. 10.1371/journal.pone.0161579 27559744 PMC4999091

[138] Khojasteh SB Villar JR Chira C González VM De La Cal E. Improving fall detection using an on-wrist wearable accelerometer. Sensors. (2018) 18(5):1350. 10.3390/s18051350 29701721 PMC5982860

[139] Broadley RW Klenk J Thies SB Kenney LPJ Granat MH. Methods for the real-world evaluation of fall detection technology: a scoping review. Sensors. (2018) 18(7):2060. 10.3390/s18072060 29954155 PMC6068511

[140] Brull Mesanza A D’Ascanio I Zubizarreta A Palmerini L Chiari L Cabanes I. Machine learning based fall detector with a sensorized tip. IEEE Access. (2021) 9:164106–17. 10.1109/ACCESS.2021.3132656

[141] Pang I Okubo Y Sturnieks D Lord SR Brodie MA. Detection of near falls using wearable devices: a systematic review. J Geriatr Phys Ther. (2019) 42(1):48–56. 10.1519/JPT.0000000000000181 29384813

[142] Vandenberg AE Van Beijnum B-J Overdevest VGP Capezuti E Johnson TM. US and Dutch nurse experiences with fall prevention technology within nursing home environment and workflow: a qualitative study. Geriatr Nurs (Minneap). (2017) 38(4):276–82. 10.1016/j.gerinurse.2016.11.005 PMC6546295 27956058

[143] Okubo Y Schoene D Lord SR. Step training improves reaction time, gait and balance and reduces falls in older people: a systematic review and meta-analysis. Br J Sports Med. (2017) 51(7):586–93. 10.1136/bjsports-2015-095452 26746905

[144] Agrawal DK Usaha W Pojprapai S Wattanapan P. Fall risk prediction using wireless sensor insoles with machine learning. IEEE Access. (2023) 11:23119–26. 10.1109/ACCESS.2023.3252886

[145] Ponce H Martinez-Villasenor L Nunez-Martinez J. Sensor location analysis and minimal deployment for fall detection system. IEEE Access. (2020) 8:166678–91. 10.1109/ACCESS.2020.3022971

[146] Morey SA Barg-Walkow LH Rogers WA. Managing heart failure on the go: usability issues with mHealth apps for older adults. Proc Hum Fact Ergon Soc Annu Meet. (2017) 61(1):1–5. 10.1177/1541931213601496

[147] Ohta T Osuka Y Shida T Daimaru K Kojima N Maruo K Feasibility, acceptability, and potential efficacy of a mobile health application for community-dwelling older adults with frailty and pre-frailty: a pilot study. Nutrients. (2024) 16(8):1181. 10.3390/nu16081181 38674872 PMC11054015

[148] Fanning J Brooks AK Robison JT Irby MB Ford S N’Dah K Associations between patterns of physical activity, pain intensity, and interference among older adults with chronic pain: a secondary analysis of two randomized controlled trials. Front Aging. (2023) 4:1216942. 10.3389/fragi.2023.1216942 37564194 PMC10411520

[149] Spann A Stewart E. Barriers and facilitators of older people’s mHealth usage: a qualitative review of older people’s views. Hum Technol. (2018) 14:264–96. 10.17011/ht/urn.201811224834

[150] Ahmed MI Kannan G. Secure and lightweight privacy preserving internet of things integration for remote patient monitoring. J. King Saud Univ. Comput. Inf. Sci. (2022) 34(9):6895–908. 10.1016/j.jksuci.2021.07.016

[151] Taheri-Kharameh Z Malmgren Fänge A Ekvall Hansson E Bashirian S Heidarimoghadam R Poorolajal J Development of a mobile application to screen and manage fall risks in older people. Disabil Rehabil Assist Technol. (2022) 17(3):362–7. 10.1080/17483107.2020.1785562 32608287

