# Supplementary material for: Mobile health (mHealth) technologies for fall prevention among older adults in low-middle income countries: bibliometrics, network analysis and integrative review
Source: Front Digit Health. 2025 Mar 28;7:1559570. doi: 10.3389/fdgth.2025.1559570 (PMC11985854; doi:10.3389/fdgth.2025.1559570)
Supplement: Supplementary file 1 [file Table1.docx]

**Mobile Health (mHealth) Technologies for fall prevention among community-dwelling older adults in Low-Middle Income (LMIC) Countries: Bibliometrics, Network Analysis and Scoping Review**

**Database Search Strings**

| Database | Hits | Extractable | Actual |
| --- | --- | --- | --- |
| Scopus | 61 | CSV | ( ( falls OR fall ) AND ( risk OR prevent* OR system* OR manage* ) ) AND ( "Mobile Health" OR "mHealth" ) AND ( patients OR "older adults" ) |
| IEEE Xplore | 23 | CSV | ( ( falls OR fall ) AND ( risk OR prevent* OR system* OR manage* ) ) AND ( "Mobile Health" OR "mHealth" ) AND ( patients OR "older adults" ) |
| PubMed | 58 | CSV | ( ( falls OR fall ) AND ( risk OR prevent* OR system* OR manage* ) ) AND ( "Mobile Health" OR "mHealth" ) AND ( patients OR "older adults" ) |
| ACM | 129 | Citations Only | [[All: falls] OR [All: fall]] AND [[All: risk] OR [All: prevent*] OR [All: system*] OR [All: manage*]] AND [[All: "mobile health"] OR [All: "mhealth"]] AND [All: "older adults"] |
| BioMed Central | 142 | Not extractable | ( ( falls OR fall ) AND ( risk OR prevent* OR system* OR manage* ) ) AND ( "Mobile Health" OR "mHealth" ) AND "older adults" |
